# Genomic approaches in the management and treatment of breast cancer

**DOI:** 10.1038/sj.bjc.6602410

**Published:** 2005-02-15

**Authors:** J C Chang, S G Hilsenbeck, S A W Fuqua

**Affiliations:** 1 Breast Center, Baylor College of Medicine, One Baylor Plaza, Houston, TX 77030, USA

**Keywords:** microarrays, breast, prediction response, prognosis

## Abstract

Breast cancer is the most common malignancy afflicting women from Western cultures. It has been estimated that approximately 211 000 women will be diagnosed with breast cancer in 2003 in the United States alone, and each year over 40 000 women will die of this disease. Developments in breast cancer molecular and cellular biology research have brought us closer to understanding the genetic basis of this disease. Unfortunately, this information has not yet been incorporated into the routine diagnosis and treatment of breast cancer in the clinic. Recent advancements in microarray technology hold the promise of further increasing our understanding of the complexity and heterogeneity of this disease, and providing new avenues for the prognostication and prediction of breast cancer outcomes. The most recent application of microarray genomic technologies to studying breast cancer will be the focus of this review.

Mortality from breast cancer results from the ability of some tumours to metastasise to distant sites. Selecting patients with micrometastases at diagnosis is crucial for clinicians in deciding who should and who should not receive toxic and expensive adjuvant chemotherapy to eradicate these metastatic cells. Axillary nodal status, the best marker now available, is still an imperfect indicator, since about 25% of node-negative patients do harbour micrometastases and are destined to recur, while up to 25% of node-positive patients will not recur even without adjuvant treatment after many years of follow-up. In spite of extensive studies, expression of most individual genes has not proven powerful enough for routine clinical use to predict accurately distant metastases over the lifetime of an individual patient. However, recent developments in applying microarray technologies to breast tumour samples suggest that these new techniques may provide for the transition of molecular biological discoveries to clinical application, and will generate clinically useful genomic profiles that more accurately predict long-term outcome of individual breast cancer patients.

## NATURAL HISTORY OF BREAST CANCER

Breast cancer is characterised by a very heterogeneous clinical course. A major goal of recent studies is to determine whether RNA microarray expression profiling, or DNA array gene amplification/gene loss patterns, can accurately predict an individual's long-term potential for recurrence from breast cancer, so that appropriate treatment decisions can be made. It is well established that some aspects of breast cancer heterogeneity is related to the different risk factors for diagnosis of this disease, such as race, diet, age, environmental factors, and cumulative exposure to the sex hormone oestrogen. The diversity in clinical course of breast cancer is undoubtedly related to differences in tumour growth rates, tumour invasiveness, metastatic potential, and other complex cellular growth signalling and survival pathways. It has long been held that knowledge of these various biological factors would help individualise patient treatment, so that patients could be classified into subsets with varying risks of recurrence. The reality, however, is that after 20 years of searching for new single factors, we still have very few biomarkers that accurately prognosticate breast cancer disease-free or overall survival in the absence of treatment (prognostic factors), or that predict response to particular therapies (predictive factors). The standard prognostic factors currently used for primary breast cancer decision making in the United States (reviewed in Clark, 2001) are: involved axillary node status (Fisher *et al*, 1978), histologic subtype, tumour size (Carter *et al*, 1989), nuclear grade (Fisher *et al*, 1980), oestrogen and progesterone receptor (ER and PR) status (McGuire, 1980), and measures of cellular proliferation (Clark *et al*, 1989). A number of factors useful for prediction of treatment outcomes have also been put into routine clinical practice. These include: ER, PR, and HER-2/c-Erb-B2. Although many genes were originally attractive biomarkers with appropriate biologic rationale, they have failed to improve independently our prediction of outcome when compared to these standard factors. In addition, while combinations of standard prognostic factors can identify subsets of patients with highly significantly different disease survival curves, they still predict individual outcomes poorly. Thus, few molecular markers discovered during the current revolution in breast cancer molecular biological studies have come into clinical use as standard prognostic or predictive factors. In addition, the role of prognostic factors in the management of breast cancer has clearly changed, with the majority of node-negative patients now undergoing systemic adjuvant therapy because we cannot precisely determine an individual's risk of recurrence. Undoubtedly, since only a minority of node-negative patients will ever develop a recurrence, there is a critical need to identify those patients with extremely low risks of breast cancer recurrence to spare those patients unnecessary overtreatment of their disease.

## THE APPLICATION OF MICROARRAY TECHNOLOGIES TO BREAST CANCER

RNA expression of individual genes can be detected and quantified by a variety of techniques, such as Northern blot analysis, S1 nuclease protection, differential display, and serial analysis of gene expression or SAGE. Recently, two array-based technologies, cDNA and oligonucleotide arrays, have been applied to gene expression quantification. Simply defined, a microarray is an orderly arrangement of known and est (expressed sequence tag) DNA samples attached to a solid support that can be interrogated with cDNA or genomic DNA. The advantage of the newer microarray technologies is the ability to measure the RNA expression of thousands of genes at one time, and to relate how the gene expression pattern of one gene correlates to the expression of other genes in or between different tumour samples, or to measure DNA amplification or loss of DNA. The simplicity of experimental design for microarray analysis provides a vehicle to tackle the complex nature of the breast cancer genome with exquisite detail. However, emerging from our early experience with this technology, there is a growing appreciation that ‘more data’ are not necessarily better without attention to study design. Experimental design issues will be the subject of a later section.

Since the RNA expression microarray technology provides a method for monitoring the RNA expression of many thousands of human genes at one time, there was considerable anticipation that it would quickly and easily revolutionise our approaches to cancer diagnosis, prognosis, and treatment. The reality remains extremely promising but is also complex. A potential complication in the application of microarray technology to primary human breast tumour samples is the presence of variable numbers of normal cells, such as stroma, blood vessels, and lymphocytes, in the tumour. Indeed, it has been demonstrated using gross analysis of human breast cancer specimens compared with breast cancer cell lines that the tumours expressed sets of genes in common not only with these cell lines but also with cells of hematopoietic lineage and stromal origin (Perou *et al*, 2000). Laser capture microdissection has also been successfully used to isolate pure cell populations from primary breast cancers for array profiling (Sgroi *et al*, 1999). In this seminal paper, Sgroi *et al* (1999) utilised laser capture microdissection to isolate morphologically ‘normal’ breast epithelial cells, invasive breast cancer cells, and metastatic lymph node cancer cells from one patient, and was able to demonstrate the feasibility of using microdissected samples for array profiling, as well as following potential progression of cancer in this patient. However, with the emerging data supporting important roles for the surrounding stroma in breast cancer progression, and the labour-intensive and technically challenging nature of laser capture technology with subsequent amplification of RNA for quantitation, most published investigations to date have evaluated total gene expression to identify prognostic profiles, as will be described in the next section.

## EXPRESSION MICROARRAY ANALYSES FOR THE IDENTIFICATION OF PROGNOSTIC FACTORS

Many of the first explorations into the use of expression microarrays were designed to evaluate the technology for molecular and/or morphologic phenotyping of breast tumours. One of the first comprehensive attempts to characterise the variation in gene expression between sporadic breast tumour samples was published by Perou *et al* (1999, 2000). This ground-breaking study was the first to establish that tumours could be phenotypically classified into subtypes distinguished by differences in their expression profiles. Perou *et al* examined 40 breast tumours, and 20 matched pairs of samples before and after doxorubicin treatment in their study; tumour samples were grossly dissected. An ‘intrinsic gene set’ of 476 cDNAs were selected that were more variably expressed between the 40 sporadic tumours than between the paired samples. This intrinsic gene set was then used to cluster and segregate the tumours into four major subgroups: (1) a ‘luminal cell-like’ group expressing the ER, (2) a ‘basal cell-like’ group expressing keratins 5 and 17, integrin*β*4, and laminin, but lacking ER expression, (3) an Erb-B2-positive subset, and (4) a ‘normal’ epithelial group.

A subsequent study by this group has extended the molecular profiling of breast cancer by applying their intrinsic gene set to cluster 78 cancers (the tumours from their previous study were included in these), three fibroadenomas, and four normal breast tissue samples (Sorlie *et al*, 2001). The same subgroups were found as before (Perou *et al*, 1999, 2000), except the luminal, ER-positive group that subdivided into further subsets with distinctive gene expression profiles. Since clinical outcomes were available on some of the patients, the authors also examined whether their phenotypic profiles could function as prognostic factors. Univariate survival analysis was performed on 49 patients from the study with locally, advanced disease, but without evidence of distant metastasis. Although ER positivity was not a significant prognostic factor on its own in this analysis, the luminal-type group enjoyed a more favourable (Sorlie *et al*, 2001) compared to the other groups. Conversely, the basal-like group had a significantly poorer prognosis. This study is clearly encouraging that significant differences in outcome can be ascertained from microarray expression profiling.

However, some limitations with this molecular classification exist. With the statistical methods (i.e. hierarchical clustering) used in this study, new cancers cannot be assigned to a particular molecular group. Assignment of a sample in hierarchical clustering algorithms to different branches of the dendogram is dependent on the selected sets of genes and the type of clustering used (central linkage, complete linkage, etc.). With each new case that is added to the data, the dendogram branches get reorganised and therefore this methodology cannot be used prospectively to classify new cases. In addition, the independent prognostic value of the molecular classification is currently unknown. The molecular classification in this study did not include the current clinical parameters like tumour grade, steroid receptor status, and HER-2/*neu.* In essence, there may be more clusters and molecular subtypes of breast cancer that may be apparent if larger sample sets are available (McShane *et al*, 2002). Such formal statistical testing has not yet been carried out on the current molecular classification.

More recently, van't Veer *et al* (2002b) have used RNA expression microarray analyses to identify a 70 gene prognostic signature (‘classifier’) in young, axillary lymph node-negative patients using a training set of 78 tumours, and then tested the classifier in a validation set of 19 tumours. The study used a case/control design and employed 5 years of clinical follow-up to define their ‘good’ (controls) *vs* ‘poor’ (cases) prognosis patients. The optimally accurate prognostic classifier correctly predicted disease outcome for 65 out of the 78 (83%) patients, identify poor prognosis outcomes with a sensitivity of 85%, and good outcomes with a specificity of 81%. Thus, the study demonstrates the feasibility of molecular profiling for subclassification of patient outcomes using undissected clinical material.

van de Vijver *et al* (2002) have now extended this study with 234 additional young (<53 years), stage I–II breast cancer patients with both node-negative and node-positive disease using the 70 classifier genes from the earlier study (van't Veer *et al*, 2002a) to classify the patients. The authors were able to classify patient outcomes (sensitivity=93%, specificity=53%) that are consistent, or perhaps better than estimates which can be obtained with current prognostic indices.

A few investigators have begun to study putative precursor lesions of invasive disease, such as ductal carcinoma *in situ* (DCIS), using genomic approaches. Porter *et al* (Porter & Polyak, 2003) have exploited SAGE analysis to compare two SAGE libraries prepared from DCIS to two libraries each of normal, invasive, and metastatic cancer. Of note is that the authors used either manual macrodissection or magnetic bead separation specific for epithelial cell content to prepare these libraries. They found that tumours of different histology had very distinct gene expression patterns. However, no genes seemed to be specific only for the DCIS or metastatic lesions. Interestingly, the most profound expression pattern changes were found to occur during the early normal to DCIS transition, suggesting that this type of study might identify future targets for chemoprevention.

Recently, Adeyinka *et al* (2002) have performed a systematic study comparing six cases of DCIS with necrosis to four cases without necrosis utilising manual microdissection or laser capture microdissestion to prepare the samples for microarray analysis. These authors report that only 69 genes were consistently and differentially expressed between the two histological types of DCIS lesions. Genes important for angiogenesis were notably increased in the DCIS with necrosis group of tumours, as well as other genes involved in migration and hypoxia. Thus, this study demonstrates that although gene expression is mostly similar between morphologically distinct types of neoplasia, differences in expression can be identified using expression array profiling, providing hope that this technology will provide profiles predicting cell behaviour in early breast disease. Since it has been demonstrated that very early precursor lesions, such as atypical ductal hyperplasia, are genetically related to invasive cancer, and are indeed precursor lesions (O'Connell *et al*, 1994, 1998), there is much anticipation that these lesions will provide valuable information about the origin and aetiology of early disease. However, systematic microarray analyses with ductal hyperplasias have yet to be reported, probably due to their rare inclusion in established frozen tumour banks, and their small size.

A few studies have utilised new genomic approaches for the study of inherited breast cancer. There is accumulating evidence, both epidemiological and histological, that tumours arising as a result of mutations in the two breast cancer susceptibility gene families (BRCA1 and BRCA2) are biologically distinct. For instance, BRCA1 breast cancers are most often ER and PR negative, but BRCA2 cancers more often tend to be positive for these receptors (Verhoog *et al*, 1999). In a seminal paper published by Hedenfalk *et al* (2001), seven tumours each from BRCA1 and BRCA2 gene mutation carriers, or sporadic breast cancers, were compared by expression microarray analysis. They found that the gene expression profiles of the three tumour groups differed significantly from each other, underscoring the fundamental differences between BRCA1 and BRCA2 mutation-associated tumours. Of course, a potential confounding issue was the differential distribution of ER between the BRCA1 and BRCA2 tumours. However, even after removal of ER/PR-associated genes from the analysis, the two inherited tumour groups were still discernable. Thus, ER status alone does not fully explain the observed differences in gene expression profiles. Although this study is obviously very small, and other confounding issues such as tumour stage, grade, and treatment were not able to be considered, it does set a foundation for larger validation studies to confirm differential genes, which could then provide important clues to the aetiology of inheritable breast cancer.

## EXPRESSION MICROARRAY ANALYSIS OF METASTATIC BREAST CANCER BEHAVIOUR

There is a growing understanding of the basic biology of the metastatic process and cancer metastasis is known to be an inherently inefficient process with only a subset of micrometastases persisting to form clinically evident metastases. Thus, the detection of breast cancer cells in the blood stream, or in secondary organs such as lymph nodes or bone marrow, does not always predict the ability of the primary tumour to form viable distant metastases. In order to increase the survival of breast cancer patients, an increased understanding of the key genes and mechanisms supporting metastatic behaviour of human breast cells needs to be elucidated. Although it can be argued that treatment with metastasis-targeting agents may be of limited value, metastasis prevention in the advanced disease setting may have a clinical role by preventing secondary metastases as tumours progress. Unfortunately, distant metastatic tumour samples from breast cancer patients are rarely biopsied or stored in tissue banks; thus, these tumours are a very rare resource that have infrequently been examined by microarray analyses.

The expression of several genes that have been profiled in human tumours (Perou *et al*, 2000) was found to be associated with the metastatic phenotype including mucin 1, c-Erb-B2, and thrombospondin.

## COMPARATIVE GENOMIC HYBRIDISATION (CGH) ANALYSIS OF BREAST CANCER

Array CGH uses thousands of genetically mapped genomic DNA clones (bacterial artificial chromosome) or cDNAs, which are spotted on glass and are hybridised in a manner similar to that used for microarray expression. The resolution of this technology is determined by the number of DNA clones on the array, and the physical chromosomal separation of the arrayed clones. Under optimal conditions, precise measurement of DNA copy number is possible (standard deviation of log 2 ratios are estimated to be=0.05–0.10) in both cell lines and clinical material (Snijders *et al*, 2001). This level of precision allows measurement of high-level amplification and single-copy alterations in heterogeneous ‘normal’ backgrounds, such as that common in clinical breast tumours.

A recent publication by Pollack *et al* (2002) found that RNA expression microarray analysis did indeed reveal DNA copy number changes to have a direct role in the transcriptional profiles of 44 human breast tumours, and impressively, 62% of the highly amplified genes concordantly showed moderate or highly elevated RNA expression. In summary, an extensive database of RNA expression and DNA copy number alterations have been compiled, many of which have been placed in public databases. It is hoped that concordances between these two genomic approaches will help to identify ‘driver’ genes involved in tumour progression, rather than just differential ‘consequences’ in gene expression. An ideal strategy is to identify initial gene expression profiles associated with clinical outcome, followed by the use of CGH analysis to pinpoint common regions of deletion or amplification within clinical subgroups.

## MICROARRAY ANALYSIS TO IDENTIFY PREDICTIVE BIOMARKERS

A predictive marker is defined as a biological factor, which can predict clinical outcome in treated patients. Thus, there are two types of questions that need to be addressed. First, who needs treatment? Prognostic factors are useful to identify a ‘poor prognosis’ group that could benefit from treatment. The second question is of those who need treatment, which treatment should they receive? Predictive factors would be useful to answer this later question. Systemic chemotherapy for operable breast cancer significantly decreases the risk of relapse and death (EBCTG, 1998a, 1998b). However, although these large clinical trials have confirmed the value of systemic therapy, it is not possible to identify at the outset those patients who are likely to respond to adjuvant treatment or which type of treatment should be used. Thus, there is a need to identify breast cancer patients who will benefit from specific adjuvant therapies, while sparing others from the side effects of futile treatment. Unlike patients with advanced breast cancer, in whom response can be assessed by tumour measurements after a few cycles of treatment, patients with early breast cancer have no measurable disease after primary surgery. Thus, no methods are now available to separate patients likely to respond to standard adjuvant treatment from those unlikely to benefit who may then choose more experimental treatments in the context of a clinical trial. This is because we cannot yet answer our first question, prognosis, adequately. Owing to these arguments, the accepted practice is to prescribe adjuvant chemotherapy even if the expected benefit is low (Fisher *et al*, 1998). A good example of this practice is that we give everyone with ER-positive disease tamoxifen therapy, even though we know that only 60% will respond to this treatment.

Treatment given before surgery (neoadjuvant therapy) has a number of advantages in breast cancer including earlier assessment of response to therapy, and access to the primary tumour during early treatment for *in vivo* testing for predictive markers whose expression correlates with successful treatment. Unlike response in the metastatic setting where one can measure response at metastatic sites, but cannot estimate effects on survival, response to neoadjuvant chemotherapy is a validated surrogate marker for improved survival and may be used to test the efficacy of treatment regimens. In the NSABP B-18 study, survival outcome was better in patients whose tumours responded to neoadjuvant chemotherapy compared to those who had chemotherapy-resistant disease, especially those who achieved pathologic complete response (Fisher *et al*, 1998). These data indicate that tumour response to neoadjuvant chemotherapy correlates with outcome, and the response in the primary tumour mirrors the effect of chemotherapy on micrometastases (Fisher *et al*, 1998). Likewise, in a smaller study involving 158 patients, clinical response to neoadjuvant chemotherapy was found to closely correlate with improved clinical outcome and response to neoadjuvant chemotherapy was the only independent variable associated with decreased risk of death (Chang *et al*, 1999). With neoadjuvant chemotherapy, the primary breast cancer provides a unique opportunity for assessing predictive markers and for studying hypothesis-generating relationships, in that it allows for measurements of possible biologic determinants to be made before treatment in an intact human tumour.

Studies have been conducted assessing the amount of total RNA obtained from each core biopsy of primary breast cancers undergoing neoadjuvant chemotherapy for its use in expression microarray experiments. From each core biopsy, sufficient total RNA was extracted for oligonucleotide array analysis and preliminary patterns predictive of sensitivity and resistance to specific treatments have been reported (Chang *et al*, 2003), where others report 45% (Buchholz *et al*, 2002) or as high as 93% (Ellis *et al*, 2002) of core biopsies to yield sufficient high-quality RNA for array analysis. Other investigators have reported faithful linear RNA amplification protocols using limited amounts of RNA from microdissected breast tissues (Aoyagi *et al*, 2003). Further work is essential in integrating amplification protocols into large-scale microarray analysis, and validating these pilot predictive expression patterns into independent patient cohorts.

A neoadjuvant approach was also undertaken by Buchholz *et al* (2002) to look at the effects of chemotherapy on gene expression. The authors obtained sufficient RNA from core biopsies of five patients to obtain serial microarray expression profiles. Patients with good pathological responses to neoadjuvant treatment had gene profiles that clustered distinctly from those of patients who were poor responders to treatment. Unfortunately, all the patients had different gene expression changes after chemotherapy, with no single gene expression changes significantly associated with response in all five patients. Their result could be due in part to the small number of patients examined, and the heterogeneity of treatments in this study.

**Figure 1:**
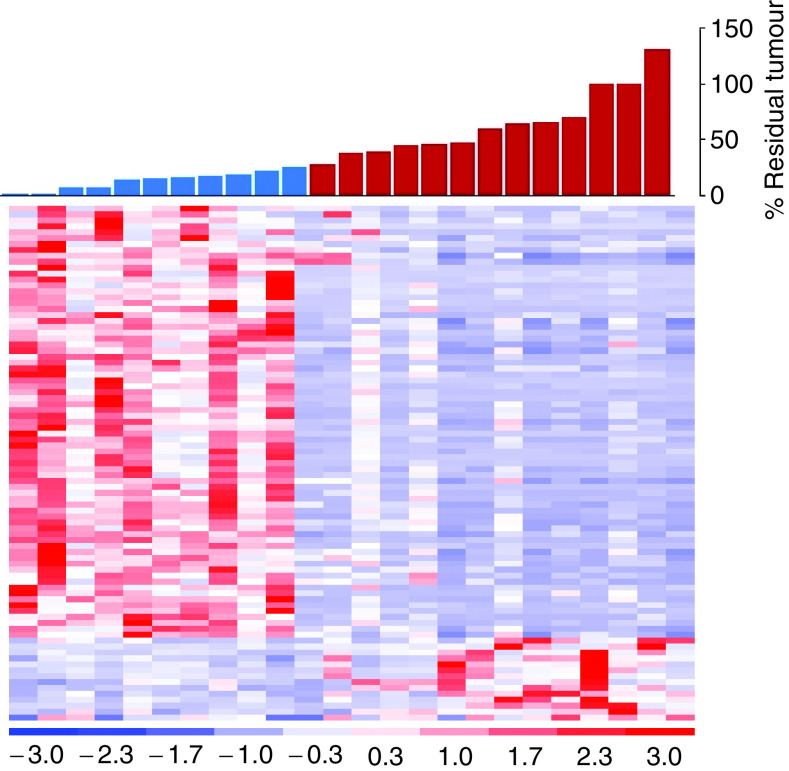
Hierarchical clustering of genes correlated with docetaxel response. Sensitive tumours (S) are defined as 25% residual disease or less (shown as blue bars), and resistant tumours (R) are defined as greater than 25% residual disease (shown as red bars). The expression levels are shown in red (expression levels above the mean for the gene) and blue (levels below the mean for the gene).

**Figure 2:**
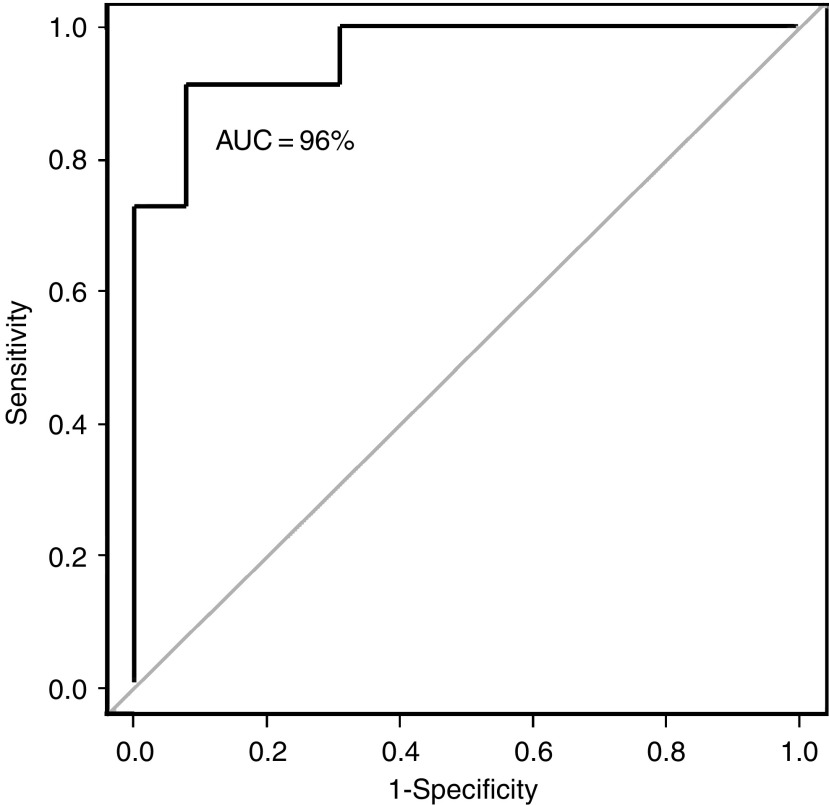
Receiver operating characteristic (ROC) curve for predicting response to docetaxel using the 92-gene classifier, with positive and negative predictive values of 92 and 83%, respectively. The area under the curve is 0.96.

More recently, we have shown that gene profiling can be used to predict accurately response to neoadjuvant docetaxel (Chang *et al*, 2003). The study enrolled 24 subjects, extracted sufficient RNA from all core-needle biopsies and constructed a 92-gene predictor of response (Figure 1). In a complete crossvalidation analysis, which gives an unbiased estimate of performance on future samples, the classifier correctly identified 10 of 11 responders and 11 of 13 nonresponders for an overall accuracy of 88% (Figure 2). Correlation between RNA expression measured by the Affymetrix arrays and semiquantitative RT–PCR was also ascertained. In addition, this classifier was validated in an independent set of six subsequent patients. We therefore have identified preliminary molecular profiles in primary breast cancers that appear to predict response or lack of response to docetaxel. This technology offers a potentially useful predictive clinical test for docetaxel sensitivity that, when validated, may reduce unnecessary treatment for women with breast cancer. In addition, these results compare very favourably with the best existing predictive factors for response to specific therapy, and strongly suggest that after appropriately extensive validation, microarray profiling will be useful for treatment selection. Additional work in ascertaining expression patterns for other commonly prescribed chemotherapy regimens, like anthracyclines and capecitibine, is underway, in the hop that these patterns differ between regimens so that predictive tests for the selection of appropriate treatment can be conducted to minimise toxicity and maximise efficacy for women with breast cancer.

A second neoadjuvant study was recently published using cDNA arrays to develop predictor for paclitaxel, fluorouracil, doxorubicin, and cyclophosphamide, involving 24 samples. A classifier with 74 markers was developed, with 78% accuracy, suggesting that transcriptional profiling has the potential to identify a gene expression pattern in breast cancer that may lead to clinically useful predictors of chemotherapy response (Ayers *et al*, 2004). Outstanding issues on the optimal method for tissue acquisition still remain. In the latter study, fine-needle aspiration was used, as compared to core-needle biopsies. Each technique has their relative advantages and limitations. In a study comparing the two techniques, both yielded a similar quality and quantity of total RNA, with similar expression profiles. The authors concluded that each technique has relative advantages. While fine-needle aspiration provided patterns representative of the tumour cell population, core-needle biopsies included patterns of stromal origin. The selection of the preferred biopsy sampling technique for gene expression arrays would be dependent on the study design, patient population, and the aims of the each individual study (Symmans *et al*, 2003).

The combined neoadjuvant treatment approaches, and expression microarray technology offers a potentially clinically useful method for developing predictive tests for chemotherapy sensitivity that, when validated, may reduce unnecessary treatment for women with breast cancer.

## EXPERIMENTAL DESIGN AND STATISTICAL ANALYSIS

As seen above, genomic approaches can address a wide range of objectives important in breast cancer. These include, for example, molecular subclassification of breast cancer, characterisation of pathways important in breast cancer aetiology and progression of premalignant lesions, and prognostication of natural history or prediction of benefit to specific therapies. The first two studies focus on discovering new classes of samples or genes, while the latter two are examples of problems in classification.

At best, genomic experiments can generate a gold mine of data that may, with proper ‘mining’, help shed light on questions far beyond those originally envisioned. At worst, without careful planning these expensive and complex experiments may fail to illuminate even their primary objectives. In all cases, it is very important to minimise possible sources of confounding factors. Samples should be handled and prepared in as identical a manner as possible. Standard methods, such as blinding of laboratory staff, and processing samples in batches that include examples of all relevant classes, is common practice in single gene studies and is even more important here.

In clinical trials, sample sizes are planned ahead of time to ensure that the number of subjects to be enrolled will be adequate to address the question. Reporting guidelines now include planned sample sizes and target effect sizes. Traditional prognostic or predictive studies are beginning to follow suit. In sharp contrast, sample sizes in most genomic (expression arrays, CGH, SAGE) experiments to date appear to have been determined by the limited number of frozen samples available and the cost of arrays. As a result, studies have tended to be very small. In the future, as studies are undertaken that propose to change clinical practice, larger samples sizes, which are more likely to encompass the full diversity of the target population, will be required. Thus, reviews for funding of such studies are beginning to require more rigorous justification.

Study objectives also determine the most appropriate methods of analysis. To date, class discovery studies have used unsupervised methods, especially cluster analysis, to ‘discover’ sample or gene groupings. Such studies are generally exploratory or hypothesis generating, and confirmation of results often relies on subsequent correlation with further supplemental biological or bioinformatic data. Analysis generally proceeds in steps, beginning with filtering of genes and samples to remove poor quality samples, and uninformative or poorly measured genes. This is followed by clustering or data mining designed to uncover ‘hidden’ groups or relationships. The ‘significance’ of such groups or relationships can be difficult to assess because any data set, even a randomly generated one, can be clustered. Fortunately, methods have been proposed to assess the stability or reliability of the clustering that may help distinguish real from spurious results. To our knowledge, there are no standard methods to determine an appropriate sample size for such studies.

Class prediction has typically been addressed with a case/control type of design (i.e. ER positive *vs* ER negative; disease free *vs* relapsed), and samples are included because of their known status. All other things being equal, the most powerful discrimination of groups is obtained when cases and controls are equally represented. Cluster analysis has sometimes been used in the analysis of such studies in the hope that groups will cluster together, but, as pointed out by Simon *et al* (2003), unsupervised cluster analysis is not effective for class comparison or class prediction. When the goal is discrimination or the selection of features that discriminate, the analysis should make use of the available information. As with class discovery, analysis begins with filtering of genes and samples to remove poor quality samples and unexpressed or poorly measured genes. Analysis then proceeds to select a subset of ‘informative’ genes, compute a score or index, and finally to define a classification rule. The process is often interative, and the score may be a simple weighted average of gene expression, as in linear discriminant analysis, or a complicated nonlinear function, as in artificial neural networks. However, the classifier is computed and the classification rule defined; it is of little value if it cannot be shown to generalise to other samples. Performance is usually assessed by the misclassification error rate, and by summary statistics borrowed from the field of diagnostic testing, such as sensitivity, specificity, and false-positive rate. ‘Resubstitution’ estimates of classification success can be computed by classifying the same cases used to create the classifier, but the estimates are biased and often highly overly optimistic. The potential for overfitting a well-known problem even in traditional single gene prognostic and predictor factor studies is simply made worse by the huge number of explanatory variables and small sample sizes.

Classifier performance is best tested by applying it to a completely new, independent set of samples. Despite some methodologic problems, the studies of van't Veer *et al* and van de Vijver (van't Veer *et al*, 2002b; van de Vijver *et al*, 2002) are ground-breaking examples. The external validation set should include all of the types of cases in the training set, and the assay process should be replicated as closely as possible.

When fully independent, external validation is not possible, then some other method, such a crossvalidation, must be used to obtain unbiased estimates of classifier performance. Properly implemented, leave-one-out crossvalidation and related methods can provide nearly unbiased estimates of classifier performance. In order for the estimates to be reliable, however, it is absolutely critical that the crossvalidation be external to the entire process by which the classifier is created (Simon *et al*, 2003). That is, in leave-one-out crossvalidation, one sample is selected to left out. The entire analysis including normalisation, expression estimation, filtering, gene selection, weighting, and classifier rule construction is performed on the remaining samples. The left-out sample is then processed and classified. The process is repeated leaving out and then classifying each sample in turn. Since each left-out case will be classified by a slightly different classifier, the resulting classification error is a nearly unbiased estimate of the classification error rate of the classifier construction process, not the error rate of a specific classifier. A final classifier is usually constructed by the same process, using all the data. Of course, independent validation is still important, especially if the training sample is relatively small because any estimates of accuracy will have wide confidence intervals. For example, in a study of 50 or fewer samples, a crossvalidated error rate of 15% will have a 95% confidence interval of 6–27%, a range far too wide to guarantee good performance on future samples. While the entire multivariable classification problem is too complex for useful sample size calculations, simpler approaches can be useful. These can be based on detecting modest differences in individual genes (gene selection phase) with good power (i.e. 80–90%) at a stringent level of significance (i.e. 0.1–1%) that will help control for multiple comparisons. Sample size should also take into account the desired width of confidence intervals for the crossvalidated or independent validation error rates.

## CONCLUSIONS

It is the goal of comprehensive, genomic-wide approaches to identify clinically useful genetic profiles that will accurately identify diagnostic subtypes, and predict prognosis and treatment responsiveness of breast cancer patients. Clearly, the management of patients would be optimised if clinicians had a molecular profile of a patient's tumour at the time of diagnosis that would accurately identify those patients who could be spared unnecessary treatment of their disease, or alternatively whose prognosis was so poor that aggressive therapies are warranted and to pinpoint the optimal therapy. It is obvious that single gene studies have to be replaced with the newer molecular approaches of microarray analysis. Undoubtedly, the benchmark for any newly identified biomarker or biomarker DNA or RNA expression profile arising from these new microarray technologies will have to be its comparison to standard prognostic factors.

The importance of experimental design to ask the appropriate question in the available data set cannot be overly stressed. Similarly, validation of generated profiles must be performed in independent data sets. The lessons we have learned in years of prognostic and predictive factor identification and implication need to be implemented in microarray approaches for the management of breast cancer. Obviously, it is hoped that this new technology will greatly improve our ability to diagnose, and predict the outcomes of breast cancer patients.
